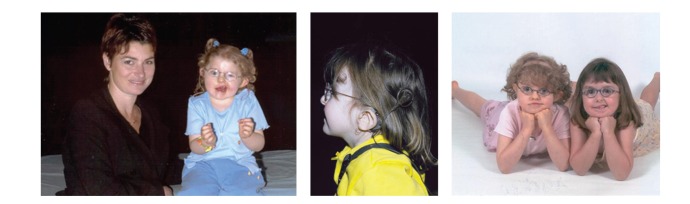# Obstructive Sleep Apnea in a Patient with CHARGE Syndrome

**DOI:** 10.1155/2012/907032

**Published:** 2012-08-28

**Authors:** Carrie-Lee Trider, Kim Blake

**Affiliations:** ^1^Dalhousie Medical School, 1459 Oxford Street, Halifax, NS, Canada B3H 4R2; ^2^Department of Pediatrics, IWK Health Centre, 5850/5980 University Avenue, P.O. Box 9700, Halifax, NS, Canada B3K 6R8

## Abstract

CHARGE syndrome is a genetic disorder characterized by choanal atresia, coloboma of the eye, and ear and cranial nerve abnormalities. We report a child with CHARGE syndrome and obstructive sleep apnea. We highlight difficulties in discerning obstructive sleep apnea-related symptoms from typical features of CHARGE syndrome. Treatment options are discussed with regard to our patient. Tonsillectomy and adenoidectomy improved physical symptoms of obstructive sleep apnea in the patient.

## 1. Introduction

CHARGE syndrome (Coloboma of the eye, Heart defects, Atresia of the choanae, Retardation of growth and development, Genital or urinary abnormalities, Ear abnormalities or deafness) is a genetic disorder that results from a mutation in the CHD7 gene on chromosome 8q12 [1]. It is diagnosed clinically by the presence of combinations of major characteristics of choanal atresia, coloboma of the eye, cup-shaped ears ± deafness, cranial nerve abnormalities, and associated temporal bone anomalies [2, 3]. The clinical phenotype of CHARGE syndrome can be very variable and ranges from mild to severe. As children get older, many of the CHARGE syndrome characteristics that affect their physical appearance become less obvious. Children with CHARGE syndrome have multiple ENT-related issues, so otolaryngologists may be the first physicians to recognize a mild spectrum of CHARGE syndrome. CHARGE syndrome is not a rare diagnosis and has a birth incidence near 1 in 8,500 [4]. It has been documented that CHARGE syndrome is often a missed diagnosis, with many patients not diagnosed until after the age of 5 [5], and therefore otolaryngologists need to be vigilant.

There is limited published literature from Hartshorne et al. [6] and Trider et al. [7] examining sleep disturbances in CHARGE syndrome. Our case was a child with CHARGE syndrome who presented with obstructive sleep apnea (OSA) symptoms.

## 2. Case Presentation

Our patient was a young male who was CHARGE syndrome gene positive. He had bilateral coloboma of the eye, characteristic ear anomalies, and lower cranial nerve dysfunction. He had choanal stenosis, which was not severe enough to require surgery. He had mild-to-moderate hearing loss and frequent ear infections with multiple sets of tubes. He also had genital hypoplasia, growth deficiency, and developmental delay. Our patient had difficulties with reflux and was tube fed. 

By the age of one, our patient was having frequent awakenings during the night and as a result was never sleeping longer than three hours at a time. His parents noted that he always snored and often had periods where he stopped breathing at night. During the day he breathed through his mouth and had problems with daytime somnolence. He had many symptoms of inattention and hyperactivity including not listening, interrupting, being easily distracted, and fidgety. Polysomnography at the age of 18 months indicated the presence of OSA. 

Continuous positive airway pressure (CPAP) was the first attempt at treatment. Compliance was the major issue since he was scared of the equipment. Tracheostomy was suggested; however his parents felt strongly that tracheostomy was not a good choice for their family. Tonsillectomy and adenoidectomy were performed without complications, and there was no further difficulty with breathing or apneas. He snored only occasionally. His parents noted that he woke up more refreshed and had more energy throughout the day. However, symptoms of inattention and hyperactivity remained. Polysomnography was not repeated in this case.

## 3. Discussion

Children with CHARGE syndrome are seen by otolaryngologists for many reasons including airway stabilization due to the choanal atresia/stenosis, sensorineural or conductive hearing loss, and difficulty in handling oral secretions, which requires Botox injections [8]. Obstructive sleep apnea is a hidden feature of CHARGE syndrome that otolaryngologists should use as an index of suspicion for the diagnosis of this syndrome. This is especially so when otolaryngologists are seeing dysmorphic patients with cranial nerve dysfunction or low, cup-shaped ears (Figure 1).

Our patient had problems with compliance with CPAP. Many children are scared of this device; moreover facial asymmetry in CHARGE syndrome can cause additional problems. These issues should be considered when recommending CPAP as a first-line treatment option. Removal of tonsilar and adenoid tissue is often considered a first-line therapy for OSA in the general pediatric population [9]. Also, postoperative airway problems have been reported as significantly decreased in children with CHARGE syndrome after tonsillectomy and adenoidectomy [10]. Therefore, the improvements in physical symptoms of OSA were expected with tonsillectomy and adenoidectomy in this patient. However, symptoms related to inattention and hyperactivity showed no improvement. This is likely related to the behavioral phenotype of CHARGE syndrome itself and is not as a result of OSA. These symptoms are not useful clinically to assess the presence or absence of OSA in the CHARGE syndrome population. Tracheostomy is a third treatment option that is often offered to children with OSA and CHARGE syndrome. The parents of this patient felt strongly that tracheostomy was not a good choice for their family. In a family caring for a child requiring extensive behavioral and medical management it is important to address caregiver concerns. 

## Figures and Tables

**Figure 1:**